# Transcranial Direct Current Stimulation Effects on Memory Consolidation: Timing Matters

**DOI:** 10.1523/ENEURO.0481-18.2019

**Published:** 2019-06-11

**Authors:** Alicia Nunez Vorobiova, Ivan Pozdniakov, Matteo Feurra

**Affiliations:** 1 School of Psychology, National Research University Higher School of Economics, Moscow 10100, Russian Federation; 2 Center for Cognition and Decision Making, Institute for Cognitive Neuroscience, National Research University Higher School of Economics, Moscow 10100, Russian Federation

**Keywords:** anodal, brain stimulation, consolidation, dorsolateral prefrontal cortex, long-term memory, tDCS

## Abstract

Transcranial direct current stimulation (tDCS) is a promising tool for modulation of learning and memory, allowing to transiently change cortical excitability of specific brain regions with physiological and behavioral outcomes. A detailed exploration of factors that can moderate tDCS effects on episodic long-term memory (LTM) is of high interest due to the clinical potential for patients with traumatic or pathological memory deficits and with cognitive impairments. This commentary discusses findings by Marián et al. (2018) recently published in *Cortex* within a broad context of brain stimulation in memory research.

## Significance Statement

Here, we discuss the recent study of Marián et al. (2018) that demonstrated a disruption of long-term retention of remote memory after application of transcranial direct current stimulation (tDCS) over the right dorsolateral prefrontal cortex (DLPFC). We address methodological issues of tDCS application such as timing, site of stimulation, electrode montage and stimulation parameters (intensity, duration). Moreover, since tDCS effects are often under debate in terms of reliability, we point at the importance of the statistical design and at the consistency with previous evidence.

## 

Recent studies questioned the reliability of transcranial direct current stimulation (tDCS) across different domains (e.g., perceptual, motor, cognitive functions) and brain networks (Horvath et al., 2015; Bestmann and Walsh, 2017). Among others, a recent meta-analysis focused on statistically non-significant close-to-zero effects of tDCS on episodic long-term memory (LTM; Galli et al., 2019). Indeed, tDCS effects on episodic memory accuracy are heterogeneous and require reevaluation, depending on different factors such as stimulation parameters and/or task specificity (Medvedeva et al., 2019).

Specifically, inside the LTM domain, it is important to consider timing of tDCS administration (Medvedeva et al., 2019) for two different reasons. First, the dynamic of tDCS effects depending on the duration and intensity of stimulation requires further clarification. Relatively low intensity (up to 1 mA) and short duration (up to 13 min) of stimulation show monotonic effects (Nitsche and Paulus, 2000, 2001), whereas longer and more intense stimulation can lead to weaker (Jamil et al., 2017) or even reversed effects (Batsikadze et al., 2013). Second, as discussed below, memory consolidation unfolds in time and is maintained by a series of processes within specific time windows. Therefore, the inconsistency of tDCS effects on LTM may be due to a temporal mismatch between the experimental intervention and the process of interest.

Consolidation is a dynamic process of memory reorganization and stabilization, which includes a complex of cellular and physiologic changes unfolding in time and resulting in transfer of labile hippocampus-dependent memories to more neocortical-dependent and stable form (Bayley et al., 2005; Squire et al., 2015). While cellular consolidation occurs within hours after memory acquisition and relies more on structural changes in the hippocampus (Morris et al., 1982), the process of system consolidation requires days and results in a redistribution of the memory engrams in the neocortex (Morris, 2006). The latter is supposed to be supported by memory reactivation or reencounter (Frankland and Bontempi, 2005) which takes place during NREM sleep (Diekelmann et al., 2009; Genzel et al., 2014) and resting wakefulness (Tambini et al., 2010). Interestingly, it has been shown that in a short period of time after the reactivation (e.g., using cues), memories are susceptible to modification or reconsolidation (Nader and Hardt, 2009), thereby more easily prone to enhancement or impairment. The study of Marián et al. (2018) extended this stream of research by addressing the tDCS modulatory effects on memory in different time windows of the consolidation process. Specifically, authors examined the impact of the right dorsolateral prefrontal cortex (DLPFC) on the reactivation process and its causal role on the delayed recall. In the study, subjects were instructed to remember word pairs (encoding phase). After a 30-min break, the reactivation phase was administered. Reactivation of encoded word pairs was induced by direct reexposure of the same pairs or by cued recall, with one word from the encoded pair presented as a cue, and the other to be recalled. Reactivation was preceded (experiment 1) or followed (experiment 2) by anodal tDCS of the right DLPFC. Finally, 7 d after the encoding phase, subjects performed the final memory test in the form of a cued recall task. The final test revealed a decrease of memory recall accuracy only in the case when tDCS was applied before but not after memory reactivation (i.e., in experiment 1). Crucially for the experiment 1, while the cued recall accuracy in the reactivation task (short-term recall) was unaffected by tDCS, its detrimental effect appeared in 7 d (long-term recall). In contrast, for the experiment 2, both short-term (reactivation) and long-term recall were unaffected by stimulation. This finding seems to suggest that the increase of prefrontal activity during memory reactivation (triggered by a cue) induces a redundant input to the hippocampus which interferes with the ongoing processes of reconsolidation (Marián et al., 2018).

In summary, this evidence shows that manipulation of the DLPFC activity is effective inside a certain time window, i.e., before but not after memory reactivation. Moreover, this effect cannot be observed immediately after tDCS application (reactivation) but only after 7 d (delayed recall). This suggests that design of tDCS experiments should follow the temporal dynamics of the process of interest (i.e., in this case, episodic memory). However, despite the high potential of the general idea behind this study, there are several weak points that hamper interpretation and further implication of results.

Despite the quite intense stimulation (yet inside the existing safety guidelines; Antal et al., 2017) and relatively large sample size, the statistical design is controversial. On the one hand, the ANOVA showed a significant main effect of stimulation on recall accuracy for experiment 1. Of note, despite the interaction between the two factors, “stimulation” and “reencounter type” (i.e., reactivation task), was not significant, the authors reported significant *post hoc* comparisons.

In addition to the ANOVA, the authors performed a multiple regression analysis which showed a significant contribution of stimulation type to the recall accuracy. However, the statistical analysis was performed separately for each experiment making difficult to disentangle reliable tDCS effects. Indeed, the baseline performance (i.e., sham) in both the experiments looks to be different: the “decreased” performance for anodal condition of the experiment 1 seems equivalent to the one of anodal and sham conditions of the experiment 2. Therefore, one might interpret the significant effect reported in the experiment 1 as an increase of performance by sham rather than a decrease by anodal stimulation.

Besides the statistical analysis, there are three more issues that might have undermined the authors’ findings. First, the authors’ rationale to apply stimulation to the right DLPFC rather than the left is unclear. Several studies revealed that verbal memory is left-lateralized (Badre and Wagner, 2007; Spaniol et al., 2009). Though another strand of researchers demonstrated encoding-retrieval hemispheric asymmetry, implying predominant involvement of the left PFC into encoding of verbal material while the right PFC into retrieval (Cabeza and Nyberg, 2000; Sandrini et al., 2003; Innocenti et al., 2010; Medvedeva et al., 2019). Thus, considering the verbal nature of the task and the similarity between processes of encoding and reconsolidation (Javadi and Walsh, 2012), it is likely that a stronger effect could pop-out if the stimulation would have been applied on the left DLPFC.

**Figure 1.**
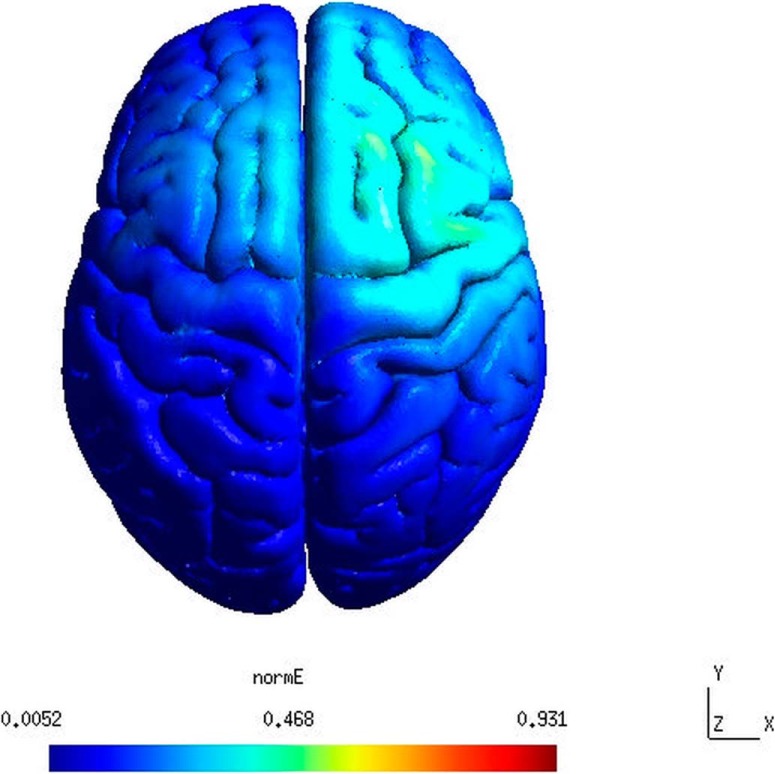
A realistic model of distribution of the tDCS-induced normalized electric field (normE) in the brain. The electric field modeling was based on the montage used in the experiment by Marián et al. (2018): anode is located on F4 and cathode is located on Cz according to the International 10–20 EEG system. The greatest amplitude of the electric field was mostly revealed on the posterior left superior frontal gyrus and posterior left middle frontal gyrus, i.e., nearby FC4 position. The electric field distribution was computed using a realistic finite element model as implemented in SimNIBS 2.1 free software (Thielscher et al., 2015).

Second, a crucial point of discussion regarding most of the tDCS studies is the electrode montage. Among different montages, tDCS is classically delivered by adopting bipolar or monopolar montages. The former implies an “active” (either cathode or anode) and a “reference” electrode placed on the scalp surface, while the latter uses a reference placed on an extracephalic target (e.g., shoulder, arm or cheek; Nasseri et al., 2015). In the study of Marián et al. (2018) a bipolar montage was applied, with the anode on F4 and the cathode on Cz according to the 10–20 EEG coordinate system. Thereby, it must be taken into account that while the target electrode was delivering anodal current to the right PFC, the reference electrode on Cz was delivering cathodal current on precentral regions thus running the risk of involving adjacent cortical areas such as the superior parietal lobule commonly involved in attention and working memory (WM) processes (Wang et al., 2015) and the right precentral gyrus involved in encoding of verbal information (Baker et al., 2001). However, a modeling approach could clarify the direction of the electrical current by taking into account different stimulation parameters such as electrode position, size, shape and current intensity. For example, according to a recent modeling tool which takes into account the electrical current spread on the cortex based on the aforementioned parameters (Thielscher et al., 2015), one may argue that the real distribution of electric field could differ from the one supposed by the authors (Fig. 1). On the one hand, the modeled electric field involves partly the right prefrontal area, meaning that the authors could eventually succeed in stimulation of their target region. On the other hand, according to the model the actual optimal hotspot (maximum intensity of the estimated electric field) of the stimulation could have been shifted slightly backward nearby FC4 electrode. This would imply a stimulation of a cortical area which is also associated to WM maintenance and attention rather than purely LTM processes (Owen et al., 2005) which could interfere with the target process. Finally, this study reveals an interesting discrepancy with previous evidence (Javadi and Cheng, 2013) which demonstrated an increase of episodic memory performance after reactivation phase combined with excitatory DLPFC stimulation. Indeed, Javadi and Cheng (2013) used anodal tDCS over the left DLPFC during memory reactivation and found an improvement of recognition performance after a 5-h interval. As noted by Marián et al. (2018), this variance of results can be explained by taking into account different encoding-retrieval intervals and task specificity (recall vs recognition).

However, the interpretation suggested by authors (i.e., activation of the right DLPFC during reconsolidation period resulted in a negative effect on memory recall) might imply that inhibition of the right DLPFC would result in a reversed (facilitatory) effect on memory recall. Interestingly, this suggestion is partly in line with findings by Sandrini et al. (2013). They applied 1-Hz repetitive transcranial magnetic stimulation (rTMS) over the right DLPFC after memory reactivation induced by a spatial-context cue. This resulted in an increase of memory recall the day after reactivation. Since 1-Hz rTMS is considered as a standard protocol for inhibition of brain activity (Chen et al., 1997; Muellbacher et al., 2000), this evidence may be considered as a reversed effect of stimulation, so that inhibition of the right DLPFC resulted in better memory performance. To be more clear, while inhibition of the right DLPFC by TMS increases memory recall, facilitation induced by tDCS of right DLPFC decreases it (Marián et al., 2018), suggesting an involvement of the same memory mechanism. However, in the study by Sandrini et al. (2013), stimulation occurred after reactivation, while in Marián et al. (2018) stimulation was effective only when was applied before but not after memory reactivation. This raises the question on the authors’ conclusion that the right DLPFC activity is critical during reactivation per se but not during reconsolidation period after reactivation. Nonetheless, this discrepancy could be due to methodological differences between these two studies (tDCS vs TMS, 5 d of retention vs 1 d, cued recall vs free recall, reactivation of encoded information vs reactivation of its context, etc.). Further systematic studies including cathodal stimulation of the DLPFC could reveal a polarity-dependent effect and replicate the TMS effect found by Sandrini et al. (2013). Moreover, this would allow examination of the time window of the right DLPFC role in memory reconsolidation process with the same method as in the present study, allowing a more reliable comparison of results. Another future research direction could also use time-locked single-pulse targeted TMS of the DLPFC in a specific time window of interest. Supposedly, this would allow for inducing selective reactivation of distinct memories, as it was demonstrated for “latent working memories” (Rose et al., 2016), thereby modulating their consolidation. Additionally, simulation of the electric field distribution on the cortex based on electrode position, size, shape and current intensity would help to obtain a predictive model of the tDCS effects (Thielscher et al., 2015).

In summary, results reported in the Marián et al. (2018) do not clearly demonstrate effects of excitatory stimulation on episodic memory consolidation. This raises important issues about the use of tDCS in memory studies. Several factors that may have influenced the reliability of the study have to be taken into account, such as the statistical design, the electrode montage and the timing of stimulation. The latter has been shown to have a particular impact for the use of tDCS in LTM investigations (Medvedeva et al., 2019).

## Synthesis

Reviewing Editor: Morgan Barense, University of Toronto Decisions are customarily a result of the Reviewing Editor and the peer reviewers coming together and discussing their recommendations until a consensus is reached. When revisions are invited, a fact-based synthesis statement explaining their decision and outlining what is needed to prepare a revision will be listed below. The following reviewer(s) agreed to reveal their identity: Domenica Veniero.

This commentary discusses the findings of a paper recently published in Cortex, Marian et al 2018, aimed at testing the effects of brain stimulation (anodal tDCS) on memory consolidation.

We found the commentary to generally be well written and we agree with most of the problems the authors highlight here. I also believe that it is important to have a critical and constructive discussion about brain stimulation effects (or lack of).

We have just a few comments:

We agree that the crucial limitation of Marian's paper is the statistics. However, in the commentary it is reported that the stimulation had no effects in either experiments. We don't think this is correct, though. In the first experiment, they found a main effect of stimulation as indicated in the paragraph 2.2.2. “Final test phase: long-term recall” (STIMULATION, F (1, 64) = 6.422, p < .05). Specifically they found a decrease in recall success when anodal tDCS was applied, but this effect was independent from the type of task used in the reencounter phase. On the other hand, we would agree that figure 2 is misleading and most importantly wrong because it is showing reencounter type * stimulation interaction, which is not significant. Please note that despite stating that “the interaction between the two factors was not significant, F (1, 64) = .000, p > .999)”, they proceed with follow-up tests.

The main problem, as reported in the commentary, is the clear difference in the sham/baseline in the two experiments, whereas it is clear that the anodal conditions are very similar. We would suggest making this the central point of the controversial statistics paragraph because, in our point of view, it invalidates the results of the first experiment.

After stating that there is no tDCS effect whatsoever, we find the last sentence quite out of place: “In summary, the study by Marián and colleagues (2018) demonstrated an interesting effect of excitatory stimulation on episodic memory consolidation, providing yet another evidence that timing matters for both LTM studies and evaluation of tDCS effects”. We were expecting to convey the fact that the statistical approach may prevent us from drawing any conclusion about tDCS and memory consolidation/timing. We doubt that the anodal stimulation had different effects in the 2 experiments (no timing effect?).

The description of the time course of the experiment on page 4 was very hard to follow. To examples are provided below, but we suggest re-writing to make this critical aspect of their experiment clearer.

- “Crucially, while the cued recall accuracy was unaffected by online tDCS”. tDCS was never performed online.

- “Reencounter was preceded (Experiment 1) or followed (Experiment 2) by anodal tDCS of the right DLPFC (reencountering phase)”. Not sure, but I suggest deleting the last parenthesis, it does not make sense to me.

The paragraph beginning with “Interpretation of the results seems controversial too” is over two pages long. We suggest breaking it up and providing clearer signposting/organization as to the ideas under discussion.

The results don't seem in line with Sandrini et al. (2013) at all. The rationale is different and so are the results and explanation.

Please rephrase: “after a break inside the same day”.

## Author Response

Dear Editor,

Thanks for giving us the opportunity to improve the Manuscript.

Please see below the point-by-point reply to Reviewers' concerns.

Looking forward for a positive feedback,

Best Regards,

The Authors

Point-by-point replies:

Reviewers: We agree that the crucial limitation of Marian's paper is the statistics. However, in the commentary it is reported that the stimulation had no effects in either experiments. We don't think this is correct, though. In the first experiment, they found a main effect of stimulation as indicated in the paragraph 2.2.2. “Final test phase: long-term recall” (STIMULATION, F (1, 64) = 6.422, p < .05). Specifically they found a decrease in recall success when anodal tDCS was applied, but this effect was independent from the type of task used in the reencounter phase. On the other hand, we would agree that figure 2 is misleading and most importantly wrong because it is showing reencounter type * stimulation interaction, which is not significant. Please note that despite stating that “the interaction between the two factors was not significant, F (1, 64) = .000, p > .999)”, they proceed with follow-up tests. The main problem, as reported in the commentary, is the clear difference in the sham/baseline in the two experiments, whereas it is clear that the anodal conditions are very similar. We would suggest making this the central point of the controversial statistics paragraph because, in our point of view, it invalidates the results of the first experiment.

Authors: We thank the Reviewer for rising up this point and we apologize we missed this information. Actually it is true that stimulation had a main effect. However, considering that a) interaction with the type of task was not significant, meaning the mere main effect of “Stimulation” did not give a clue if tDCS had specific impact on a target process respect to another one (reencounter via re-exposure vs. cued recall). Indeed, the Figure 2 is misleading since it shows interaction between the main factors that is not significant. b) Indeed, it can be that since only sham and anodal tDCS were used (in absence of control stimulation condition or control site stimulation), the effects of stimulation may be due to neurosensory side effects (e.g. scalp sensation, itching etc.), although its' effects are mainly proven on RTs (Fertonani et al., 2015; Raco et al., 2014). c) Given that the performance among the two experiments is similar for anodal stimulation while only sham differs, it might be that in the Experiment 1 there was an increase of performance due to sham stimulation rather than a decrease due to anodal stimulation. We have put a particular stress on point a) and c) in the new version of the Manuscript with a specific remark to the last point.

Reviewers: After stating that there is no tDCS effect whatsoever, we find the last sentence quite out of place: “In summary, the study by Marián and colleagues (2018) demonstrated an interesting effect of excitatory stimulation on episodic memory consolidation, providing yet another evidence that timing matters for both LTM studies and evaluation of tDCS effects”. We were expecting to convey the fact that the statistical approach may prevent us from drawing any conclusion about tDCS and memory consolidation/timing. We doubt that the anodal stimulation had different effects in the 2 experiments (no timing effect?).

Authors: We understand the Reviewer point and after careful revision of the Manuscript and of the Marián et al. (2018) paper, we agree to maintain the logic of the discussion. Therefore, we rephrased that part inside the new version of the Manuscript by summarizing the lack of statistical evidence, the similar effects of anodal stimulation between the two experiments, the lack of between-factors interaction and the lack of a control site or control condition of stimulation (e.g. cathodal) which could have controlled the difference between sham and anodal.

Reviewers: The description of the time course of the experiment on page 4 was very hard to follow. To examples are provided below, but we suggest re-writing to make this critical aspect of their experiment clearer.

- “Crucially, while the cued recall accuracy was unaffected by online tDCS”. tDCS was never performed online.

- “Reencounter was preceded (Experiment 1) or followed (Experiment 2) by anodal tDCS of the right DLPFC (reencountering phase)”. Not sure, but I suggest deleting the last parenthesis, it does not make sense to me.

Authors: We have modified this section in the new version of the Manuscript, following Reviewer's remarks.

Reviewers: The paragraph beginning with “Interpretation of the results seems controversial too” is over two pages long. We suggest breaking it up and providing clearer signposting/organization as to the ideas under discussion.

Authors: We thank the Reviewer for raising this point and we have rephrased this paragraph by splitting it into three parts (e.g. firstly…, secondly…, finally…).

Reviewers: The results don't seem in line with Sandrini et al. (2013) at all. The rationale is different and so are the results and explanation.

Authors: We agree with the Reviewer that the rationale of Sandrini et al. (2013) study is different. Moreover previous results adopting inhibitory rTMS protocol over the rDLPFC go in a different direction with respect to the ones of Marián and colleagues (2018), showing an immediate increase of memory performance (Turriziani et al., 2012). However, interpretation suggested by authors (i.e. activation of the right DLPFC during reconsolidation period resulted in a negative effect on memory recall) might imply that inhibition of the right DLPFC would result in a reversed (facilitatory) effect on memory recall. Interestingly, this suggestion is partly in line with findings by Sandrini and colleagues

(2013). They applied 1 Hz repetitive transcranial magnetic stimulation (rTMS) over the right DLPFC after memory reencounter induced by a spatial-context cue. This resulted in an increase of memory recall the day after reencounter. Since 1 Hz rTMS is considered as a protocol for inhibition of brain activity (Chen et al., 1997; Muellbacher et al., 2000), this evidence may be considered as a reversed effect of stimulation, so that inhibition of the right DLPFC resulted in better memory performance. To be more clear, while inhibition of the right DLPFC by TMS increases memory recall, facilitation induced by tDCS of right DLPFC decreases it (Marián et al., 2018) suggesting an involvement of the same memory mechanism. We decided to integrate this part to clarify comparison of the results from Sandrini et al. (2013) and Marián et al. (2018). It is not mentioned in the Sandrini et al. (2013) article that 1 Hz rTMS is considered as an inhibitory protocol. Thus we emphasized it. In addition, we rephrased this part to make it more clear to the readers.

Reviewers: Please rephrase: “after a break inside the same day”.

Authors: Rephrased to “after a 30-min break”.

References

Fertonani A, Ferrari C, Miniussi C (2015) What do you feel if I apply transcranial electric stimulation? Safety, sensations and secondary induced effects. Clin Neurophysiol 126:2181–2188.

Marián M, Szollosi Á, Racsmány M (2018) Anodal transcranial direct current stimulation of the right dorsolateral prefrontal cortex impairs long-term retention of reencountered memories. Cortex 108:80–91.

Raco V, Bauer R, Olenik M, Brkic D, Gharabaghi A (2014) Brain Stimulation Neurosensory Effects of Transcranial Alternating Current Stimulation. Brain Stimul 7:823–831.

Sandrini M, Censor N, Mishoe J, Cohen LG (2013) Causal Role of Prefrontal Cortex in Strengthening of Episodic Memories through Reconsolidation. Curr Biol 23:2181–2184.

Turriziani P, Smirni D, Zappalà G, Mangano GR, Oliveri M, Cipolotti L (2012) Enhancing memory performance with rTMS in healthy subjects and individuals with Mild Cognitive Impairment: the role of the right dorsolateral prefrontal cortex. Front Hum Neurosci 6:1–8.

## References

[B1] Antal A, Alekseichuk I, Bikson M, Brockmöller J, Brunoni AR, Chen R, Cohen LG, Dowthwaite G, Ellrich J, Flöel A, Fregni F, George MS, Hamilton R, Haueisen J, Herrmann CS, Hummel FC, Lefaucheur JP, Liebetanz D, Loo CK, McCaig CD, Miniussi C, et al. (2017) Low intensity transcranial electric stimulation: safety, ethical, legal regulatory and application guidelines. Clin Neurophysiol 128:1774–1809. 10.1016/j.clinph.2017.06.001 28709880 PMC5985830

[B2] Badre D, Wagner AD (2007) Left ventrolateral prefrontal cortex and the cognitive control of memory. Neuropsychologia 45:2883–2901. 10.1016/j.neuropsychologia.2007.06.015 17675110

[B3] Baker JT, Sanders AL, Maccotta L, Buckner RL (2001) Neural correlates of verbal memory encoding during semantic and structural processing tasks. Neuroreport 12:1251–1256. 11338201 10.1097/00001756-200105080-00039

[B4] Batsikadze G, Moliadze V, Paulus W, Kuo M-F, Nitsche MA (2013) Partially non-linear stimulation intensity-dependent effects of direct current stimulation on motor cortex excitability in humans. J Physiol 591:1987–2000. 10.1113/jphysiol.2012.249730 23339180 PMC3624864

[B5] Bayley PJ, Gold JJ, Hopkins RO, Squire LR (2005) The neuroanatomy of remote memory. Neuron 46:799–810. 10.1016/j.neuron.2005.04.034 15924865 PMC1459336

[B6] Bestmann S, Walsh V (2017) Transcranial electrical stimulation. Curr Biol 27:R1258–R1262. 10.1016/j.cub.2017.11.001 29207262

[B7] Cabeza R, Nyberg L (2000) Neural bases of learning and memory: functional neuroimaging evidence. Curr Opin Neurol 13:415–421. 10970058 10.1097/00019052-200008000-00008

[B8] Chen R, Classen J, Gerloff C, Celnik P, Wassermann EM, Hallett M, Cohen LG (1997) Depression of motor cortex excitability by low-frequency transcranial magnetic stimulation. Neurology 48:1398–1403. 10.1212/wnl.48.5.1398 9153480

[B9] Diekelmann S, Wilhelm I, Born J (2009) The whats and whens of sleep-dependent memory consolidation. Sleep Med Rev 13:309–321. 10.1016/j.smrv.2008.08.002 19251443

[B10] Frankland PW, Bontempi B (2005) The organization of recent and remote memories. Nat Rev Neurosci 6:119–130. 10.1038/nrn1607 15685217

[B11] Galli G, Vadillo MA, Sirota M, Feurra M, Medvedeva A (2019) A systematic review and meta-analysis of the effects of transcranial direct current stimulation (tDCS) on episodic memory. Brain Stimul 12:231–241. 10.1016/j.brs.2018.11.008 30503376

[B12] Genzel L, Kroes MCW, Dresler M, Battaglia FP (2014) Light sleep versus slow wave sleep in memory consolidation: a question of global versus local processes? Trends Neurosci 37:10–19. 10.1016/j.tins.2013.10.002 24210928

[B13] Horvath JC, Forte JD, Carter O (2015) Quantitative review finds no evidence of cognitive effects in healthy populations from single-session transcranial direct current stimulation (tDCS). Brain Stimul 8:535–550. 10.1016/j.brs.2015.01.400 25701175

[B14] Innocenti I, Giovannelli F, Cincotta M, Feurra M, Polizzotto NR, Bianco G, Cappa SF, Rossi S (2010) Event-related rTMS at encoding affects differently deep and shallow memory traces. Neuroimage 53:325–330. 10.1016/j.neuroimage.2010.06.011 20601000

[B15] Jamil A, Batsikadze G, Kuo HI, Labruna L, Hasan A, Paulus W, Nitsche MA (2017) Systematic evaluation of the impact of stimulation intensity on neuroplastic after-effects induced by transcranial direct current stimulation. J Physiol 595:1273–1288. 10.1113/JP272738 27723104 PMC5309387

[B16] Javadi AH, Walsh V (2012) Transcranial direct current stimulation (tDCS) of the left dorsolateral prefrontal cortex modulates declarative memory. Brain Stimul 5:231–241. 10.1016/j.brs.2011.06.007 21840287

[B17] Javadi AH, Cheng P (2013) Transcranial direct current stimulation (tDCS) enhances reconsolidation of long-term memory. Brain Stimul 6:668–674. 10.1016/j.brs.2012.10.007 23137702

[B18] Marián M, Szó́lló́si Á, Racsmány M (2018) Anodal transcranial direct current stimulation of the right dorsolateral prefrontal cortex impairs long-term retention of reencountered memories. Cortex 108:80–91. 10.1016/j.cortex.2018.07.012 30142573

[B19] Medvedeva A, Materassi M, Neacsu V, Beresford-Webb J, Hussin A, Khan N, Newton F, Galli G (2019) Effects of anodal transcranial direct current stimulation over the ventrolateral prefrontal cortex on episodic memory formation and retrieval. Cereb Cortex 29:657–665. 29329367 10.1093/cercor/bhx347

[B20] Morris RGM (2006) Elements of a neurobiological theory of hippocampal function: the role of synaptic plasticity, synaptic tagging and schemas. Eur J Neurosci 23:2829–2846. 10.1111/j.1460-9568.2006.04888.x 16819972

[B21] Morris RGM, Garrud P, Rawlins JNP, O’Keefe J (1982) Place navigation impaired in rats with hippocampal lesions. Nature 297:681–683. 10.1038/297681a0 7088155

[B22] Muellbacher W, Ziemann U, Boroojerdi B, Hallett M (2000) Effects of low-frequency transcranial magnetic stimulation on motor excitability and basic motor behavior. Clin Neurophysiol 111:1002–1007. 10825706 10.1016/s1388-2457(00)00284-4

[B23] Nader K, Hardt O (2009) A single standard for memory: the case for reconsolidation. Nat Rev Neurosci 10:224–234. 10.1038/nrn2590 19229241

[B24] Nasseri P, Nitsche MA, Ekhtiari H (2015) A framework for categorizing electrode montages in transcranial direct current stimulation. Front Hum Neurosci 9:54. 25705188 10.3389/fnhum.2015.00054 PMC4319395

[B25] Nitsche MA, Paulus W (2000) Excitability changes induced in the human motor cortex by weak transcranial direct current stimulation. J Physiol 527:633–639. 10.1111/j.1469-7793.2000.t01-1-00633.x 10990547 PMC2270099

[B26] Nitsche MA, Paulus W (2001) Sustained excitability elevations induced by transcranial DC motor cortex stimulation in humans. Neurology 57:1899–1901. 10.1212/wnl.57.10.1899 11723286

[B27] Owen AM, McMillan KM, Laird AR, Bullmore E (2005) N-back working memory paradigm: a meta-analysis of normative functional neuroimaging studies. Hum Brain Mapp 25:46–59. 10.1002/hbm.20131 15846822 PMC6871745

[B28] Rose NS, LaRocque JJ, Riggall AC, Gosseries O, Starrett MJ, Meyering EE, Postle BR (2016) Reactivation of latent working memories with transcranial magnetic stimulation. Science 354:1136–1139. 10.1126/science.aah7011 27934762 PMC5221753

[B29] Sandrini M, Cappa SF, Rossi S, Rossini PM, Miniussi C (2003) The role of prefrontal cortex in verbal episodic memory: rTMS evidence. J Cogn Neurosci 15:855–861. 10.1162/089892903322370771 14511538

[B30] Sandrini M, Censor N, Mishoe J, Cohen LG (2013) Causal role of prefrontal cortex in strengthening of episodic memories through reconsolidation. Curr Biol 23:2181–2184. 10.1016/j.cub.2013.08.045 24206845 PMC3824257

[B31] Spaniol J, Davidson PSR, Kim ASN, Han H, Moscovitch M, Grady CL (2009) Event-related fMRI studies of episodic encoding and retrieval: meta-analyses using activation likelihood estimation. Neuropsychologia 47:1765–1779. 10.1016/j.neuropsychologia.2009.02.028 19428409

[B32] Squire LR, Genzel L, Wixted JT, Morris RG (2015) Memory consolidation. Cold Spring Harb Perspect Biol 7:a021766. 10.1101/cshperspect.a021766 26238360 PMC4526749

[B33] Tambini A, Ketz N, Davachi L (2010) Enhanced brain correlations during rest are related to memory for recent experiences. Neuron 65:280–290. 10.1016/j.neuron.2010.01.001 20152133 PMC3287976

[B34] Thielscher A, Antunes A, Saturnino GB (2015) Field modeling for transcranial magnetic stimulation: a useful tool to understand the physiological effects of TMS? In: Engineering in medicine and biology society (EMBC), 2015 37th Annual International Conference of the IEEE, pp 222–225. 10.1109/EMBC.2015.7318340 26736240

[B35] Wang J, Yang Y, Fan L, Xu J, Li C, Liu Y, Fox PT, Eickhoff SB, Yu C, Jiang T (2015) Convergent functional architecture of the superior parietal lobule unraveled with multimodal neuroimaging approaches. Hum Brain Mapp 36:238–257. 10.1002/hbm.22626 25181023 PMC4268275

